# Inducible nonhuman primate models of retinal degeneration for testing end-stage therapies

**DOI:** 10.1126/sciadv.adg8163

**Published:** 2023-08-02

**Authors:** Divya Ail, Diane Nava, In Pyo Hwang, Elena Brazhnikova, Céline Nouvel-Jaillard, Alexandre Dentel, Corentin Joffrois, Lionel Rousseau, Julie Dégardin, Stephane Bertin, José-Alain Sahel, Olivier Goureau, Serge Picaud, Deniz Dalkara

**Affiliations:** ^1^ Sorbonne Université, INSERM, CNRS, Institut de la Vision, F-75012 Paris, France.; ^2^ CHNO des Quinze-Vingts, INSERM-DGOS CIC 1423, F-75012 Paris, France.; ^3^ Department of Ophthalmology, Pitié-Salpêtrière University Hospital, F-75013 Paris, France.; ^4^ ESYCOM, Université Eiffel, CNRS, CNAM, ESIEE Paris, F-77454 Marne-la-Vallée, France.; ^5^ Department of Ophthalmology, The University of Pittsburgh School of Medicine, Pittsburgh, PA 15213, USA.; ^6^ Fondation Ophtalmologique Adolphe de Rothschild, F-75019 Paris, France.

## Abstract

The anatomical differences between the retinas of humans and most animal models pose a challenge for testing novel therapies. Nonhuman primate (NHP) retina is anatomically closest to the human retina. However, there is a lack of relevant NHP models of retinal degeneration (RD) suitable for preclinical studies. To address this unmet need, we generated three distinct inducible cynomolgus macaque models of RD. We developed two genetically targeted strategies using optogenetics and CRISPR-Cas9 to ablate rods and mimic rod-cone dystrophy. In addition, we created an acute model by physical separation of the photoreceptors and retinal pigment epithelium using a polymer patch. Among the three models, the CRISPR-Cas9–based approach was the most advantageous model in view of recapitulating disease-specific features and its ease of implementation. The acute model, however, resulted in the fastest degeneration, making it the most relevant model for testing end-stage vision restoration therapies such as stem cell transplantation.

## Teaser

Three distinct inducible non-human primate models of retinal degeneration were created to test safety and efficacy of therapies.

## INTRODUCTION

Retinal degeneration (RD) diseases severely compromise the quality of patients’ lives and constitute an economic burden (*1*). Extensive research efforts are focused toward developing effective novel therapies for RD such as gene and cell therapies and retinal implants (*2*). However, at present, there is a lack of good animal models to test the effectiveness and translatability of these therapies to humans. Rodents are the most commonly used models, but they lack important anatomical features such a cone-rich macula and fovea, which result in functional differences as well as differences in the manifestation of clinical features and the progression of human diseases (*3*). The nonhuman primate (NHP) retina on the other hand is anatomically very close to the human retina, including the presence of a cone-rich macula and fovea, color vision, and acuity (*4*, *5*). In addition, NHPs have the added advantage of similarities with humans regarding genetics and immunology, which would make the manifestation of symptoms and progression of the diseases closer to human patients (*6*).

For inherited genetic disorders, an ideal model would be one that carries the mutation and shows similar symptoms as in humans, such as the one generated for Parkinson’s disease (*7*). However, generating NHP models by germline transmission is a cumbersome task and is not permitted in most countries because of national ethical laws (*8*). Some NHP facility screens have identified naturally occurring mutations for macular degeneration (*9*), retinitis pigmentosa (*10*), achromatopsia (*11*), and Bardet-Biedl syndrome (*12*). However, maintenance and breeding of such NHP lines are resource intensive and not desirable because of time and monetary constraints. Some attempts have been made to circumvent these issues by generating inducible models that recapitulate certain disease features. For example, an NHP model for diabetic ocular neovascularization was generated by Adeno-Associated Virus (AAV)-mediated delivery of vascular endothelial growth factor, resulting in angiogenesis and changes in the outer nuclear layer (ONL) of the retina (*13*). Another study generated a model for glaucoma by intracameral injection of microbeads resulting in an increase in intraocular pressure—a hallmark of glaucoma (*14*). Different studies have attempted to generate models by induction of focal regions of degeneration using a laser (*15*–*18*). Degeneration has also been caused by injection of chemicals such as cobalt chloride (*17*), sodium iodate (*19*), and sodium nitroprusside (*20*). An NHP model for achromatopsia was generated by AAV-mediated CRISPR-Cas9 deletion of the *CNGB3* gene (*21*). An inducible NHP model for rod-cone dystrophy was, however, still lacking.

To address this unmet need, we used three different strategies to generate NHP models for RD. First, an optogenetic strategy was devised, wherein a photoactivatable protein called KillerRed (KR) was used to induce photoreceptor (PR) ablation in a spatially and temporally controlled manner. KR is a dimeric red fluorescent protein genetically engineered from the hydrozoan chromoprotein anm2CP, which is a gene analog of green fluorescent protein. Upon excitation with green light (540 to 590 nm), KR is activated and produces reactive oxygen species (ROS) that result in oxidative stress and eventual cell death (*22*). KR-mediated cell ablation is versatile and has been used in different animal models such as *Caenorhabditis elegans* (*23*), zebrafish (*24*, *25*), and *Xenopus laevis* (*26*). Studies have shown KR-mediated elimination of cancer cells in vitro (*27*) and tumor reduction by KR-mediated photodynamic therapy in mice (*28*, *29*). A study in the mouse retina involving AAV-mediated delivery of KR to Müller glial cells showed successful ablation of these cells, leading to retinal structural and functional anomalies upon illumination with green light (*30*).

Our second model was generated by CRISPR-Cas9–mediated disruption of the *Rhodopsin* gene in the rod PRs. It consists of a Cas9 protein coupled with a single guide RNA (sgRNA), which can be specifically designed to target genes of interest. Insertions and deletions (indels) occur when the sgRNA guides the Cas9 to the gene of interest (*31*). The CRISPR-Cas9 system has emerged as a powerful gene-editing technique and is being applied for generation of large animal models for various diseases (*32*). Rhodopsin is a G protein–coupled receptor encoded by the *RHO* gene. It is located in the membrane disks of the outer segments of the rod PRs and is an important component of the phototransduction cascade that converts light into electrical signals. Mutations in the *RHO* gene have been identified to be responsible for approximately 10% of patients with retinitis pigmentosa (RP) and account for up to 40% of the autosomal dominant RP (*33*, *34*). Rodent models such as the P347S mouse (*35*) and P23H rats (*36*) with mutations in the *Rhodopsin* gene are available and are routinely used for vision restoration studies (*37*).

Our third model involved the surgical placement of a polymer patch between the retinal pigment epithelium (RPE) and the PRs. The RPE is a monolayer of epithelial cells present at the apical end of the PR outer segments. The PRs depend on the RPE cells for two vital functions—renewal of the outer segments that are shed daily and reconversion of the visual pigments (opsins) from their 11-cis retinal to the all-trans retinal form. Thus, a barrier between the RPE and PRs causes a disruption in these vital functions—resulting in stress, accumulation of toxic by-products, eventual shortening of segments, and cell death (*38*) as previously observed in studies of epiretinal implants (*39*).

## RESULTS

### Strategy 1: Optogenetic strategy and proof of concept in rodents

**Fig. 1.**
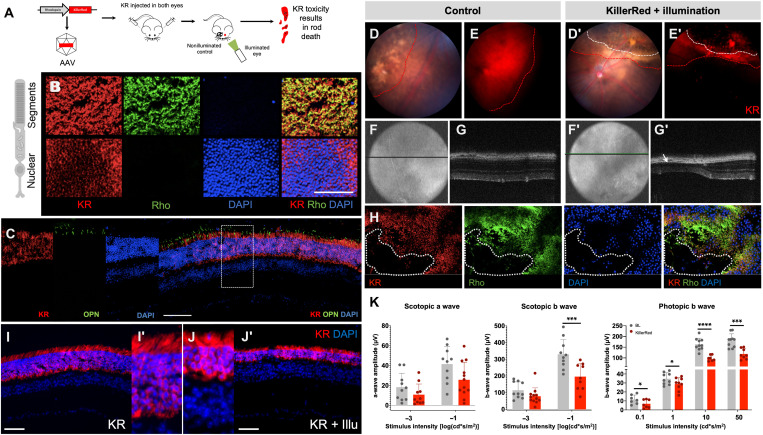
Optogenetic strategy for rod ablation and proof of concept in mouse. (**A**) Schematic representation of the experiment protocol—*KillerRed* under the control of rhodopsin promoter is packaged into AAV vectors and delivered by subretinal injection to the retina and activation of KR by light results in toxicity. (**B**) Immunolabeling of retinal flatmounts of KR-injected mice with KR (red), rhodopsin (green), DAPI (blue), and overlay of the three channels. Images are acquired at the level of the segments and the ONL. (**C**) Immunolabeling of the retinal section from the KR-injected eye with KR (red), short-wavelength opsin (green), and DAPI (blue). (**D** to **G**′) Fundus images (D and D′), fundus images in the red channel (E and E′), eye OCT (F and F′), and retinal OCT (G and G′) in KR-injected (control) (D to G) and KR-injected and illuminated mice (D′ to G′). The red dotted line demarcates the injected area, and the white dotted line demarcates the area of damage after illumination. (**H**) Immunolabeling of retinal flatmount from KR-injected and illuminated mice. Region of degeneration is demarcated by the dotted white line. (**I** to **J′**) KR immunolabeling of retina from KR-injected (KR) (I and I′) and KR-injected and illuminated mice (KR + Illu) (J and J′). (**K**) Scotopic a- and b-wave amplitudes and photopic b-wave amplitude shown as a function of stimulus intensity (*x* axis) from mice before illumination (gray bars) and 4 months after illumination (red bars); all values are shown as mean ± SD for *N* = 12 eyes. Scale bars, 50 μm. ONL, outer nuclear layer, OCT, optical coherence tomography.

The *KillerRed* gene was cloned under the control of a human rhodopsin promoter for specific expression in the rod PRs. This construct was packaged into AAV8 vector, which is known to efficiently transduce PRs in mice (*40*), and delivered by bilateral subretinal injections to wild-type (WT) (C57BL/6J) mouse retina. To induce degeneration, 8 weeks after injection, one eye was illuminated using a light-emitting diode (LED) light with an average wavelength of 565 nm. The other eye was left undilated and covered with a patch to protect from residual light and served as an injected but nonilluminated control (Fig. 1A). The intensity of the light was optimized so as to cause KR activation without light damage. We used level 4 (corresponding to approximately 5000 lux) in our system (fig. S1). Retinal flatmounts costained for rhodopsin and KR showed that KR was expressed in the segments of the rod PRs and the cell bodies (Fig. 1B). In the retinal sections, colabeling of rhodopsin with KR is observed (Fig. 1B), whereas the short-wavelength cone opsin does not costain with KR (Fig. 1C), showing that KR is expressed only in rods and not in cones. Hence, rod-specific expression of KR is achieved with our system. The expression of KR was tested by imaging the fundus in the red channel, and maximal expression was achieved between 6 and 8 weeks after injection (Fig. 1, D and E). Four weeks after illumination, loss of KR-expressing rods occurs at the illuminated area. The fundus of these mice shows distinct area of degeneration (yellow dotted line) within the injected area (white dotted line) (Fig. 1, D′ and E′). The optical coherence tomography (OCT) imaging of the both eyes revealed that the different layers of the retina remained intact in the injected control eye, whereas there was severe thinning and complete loss of the ONL in case of the illuminated eyes (Fig. 1, F to G′). Immunolabeling of the flatmount preparations of the KR-injected and illuminated retina revealed the loss of rods in the illuminated zone (Fig. 1H). KR immunolabeling of retinal sections from the control and illuminated eyes revealed that there is overall thinning of the ONL even in regions where the rods are not completely eliminated (Fig. 1, I to J′). Electroretinogram (ERG) was recorded 4 months after illumination from mice that were injected and illuminated in both eyes (Fig. 1K, red bars), and noninjected mice were used as controls (Fig. 1K, gray bars). There was a general trend of reduction in the ERG wave amplitudes under both scotopic and photopic conditions. This reduction was significant for b-wave amplitudes, whereas a-wave recordings proved difficult to measure due to high variability (Fig. 1K).

**Table 1.** Summary of injections and surgeries in NHPs. LE, left eye; RE, right eye.

Strategy 1. NHPs used for the optogenetic model					
Animal	Eye	AAV capsid	Promoter	Transgene	Dose (vg/eye)
NHP 1	RE	AAV5	hRho	KR	5 × 10^10^
NHP2	RE	AAV5	hRho	KR	5 × 10^10^
NHP 3	RE	AAV5	hRho	KR	1 × 10^11^
	LE	AAV5	hRho	KR	1 × 10^11^
NHP 4	RE	AAV5	hRho	KR	1 × 10^11^
	LE	AAV5	hRho	KR	1 × 10^11^
**Strategy 2. NHPs used for the CRISPR-Cas9 model**					
Animal	Eye	AAV capsid	Promoter	Transgene	Dose (vg/eye)
NHP5	LE	AAV5	CMV	Cas9-*Rho*	1 × 10^11^
	RE	AAV5	CMV	Cas9	1 × 10^11^
NHP6	LE	AAV5	hRho	Cas9-*Rho*	Bleb 1 (superior)—1 × 10^11^
					Bleb 2 (inferior)—1 × 10^11^
	RE	AAV5	hRho	Cas9	Bleb 1 (superior)—1 × 10^11^
					Bleb 2 (inferior)—1 × 10^11^
NHP7	LE	AAV5	hRho	Cas9-*Rho*	2 × 10^11^
	RE	AAV5	hRho	Cas9	2 × 10^11^
**Strategy 3. NHPs used for the physical model**					
Animal	Eye	Surgery			
NHP8	LE	Polyimide + parylene patch close to the fovea			
	RE	Control without patch			
NHP9	LE	Control without patch			
	RE	Polyimide + parylene patch in the superior region			

### Strategy 1: KR-mediated RD in NHP

**Fig. 2.**
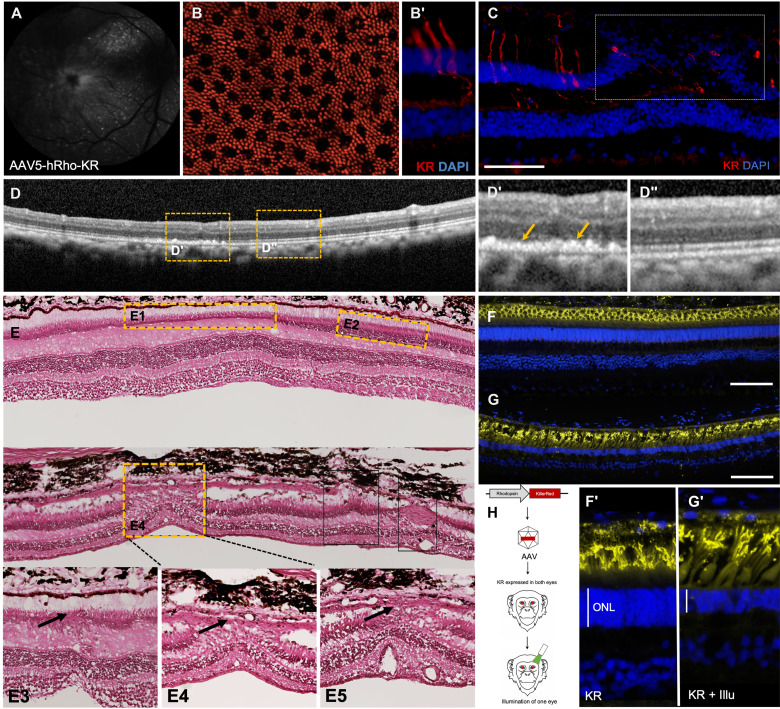
Expression of KillerRed and loss of PRs induced by illumination in NHP. (**A**) Fundus image of the KR-injected NHP eye. (**B** and **B′**) KR immunolabeling in (B) retinal flatmount and (B′) retinal section of the KR-injected eye showing rod PR-specific expression. (**C**) Immunolabeling of the KR-injected and illuminated eye. (**D** to **D″**) OCT images of the KR-injected and illuminated eye. Areas with (D′) PR loss and an adjacent (D″) control region are demarcated by the yellow dotted line and magnified in the corresponding panels. (**E**) Histological sections of the KR-injected and illuminated eye. Regions of damage are demarcated by the yellow dotted line and arrows point to degenerated areas of the ONL. Immunolabeling with rhodopsin (yellow) and DAPI for nuclear staining (blue) in (**F** and **F′**) KR-injected control eyes and (**G** and **G′**) KR-injected and illuminated eyes. (**H**) Schematic representation of the experiment protocol—KR under the control of rhodopsin promoter is packaged into AAV and delivered by subretinal injection to the NHP retina, and KR is activated by illumination with green light in one eye. Scale bars, 100 μm.

**Fig. 3.**
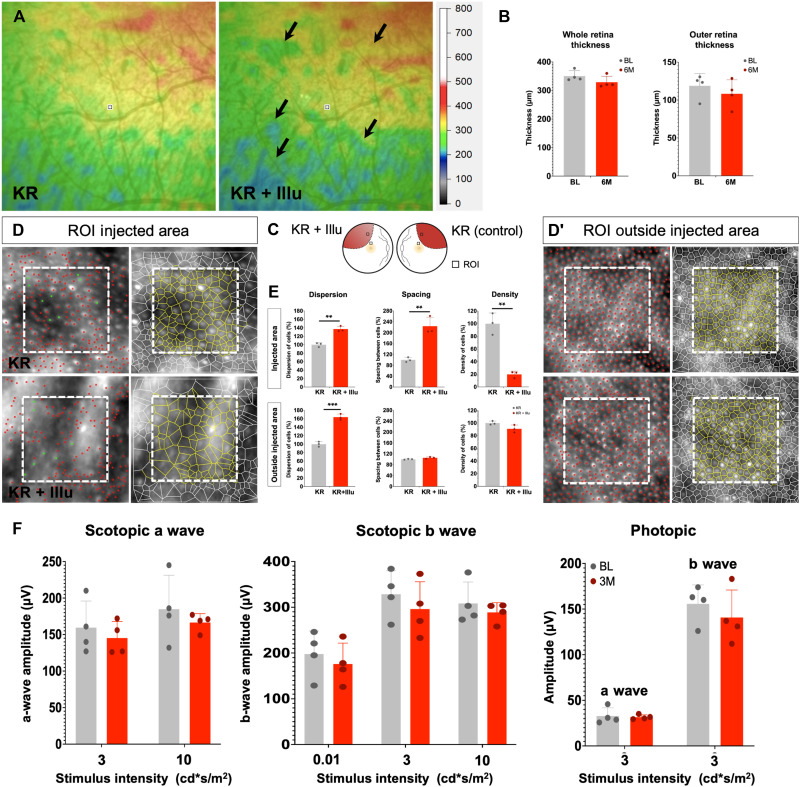
Optogene-mediated retinal degeneration in the NHP retina. (**A**) Heatmaps from the area of injection of eye injected with KR before (KR) and after illumination (KR + Illu). Arrows point toward regions of degeneration and thinner areas of the retina. (**B**) Thickness of the whole retina and the outer retina 6 months (6M) after illumination. (**C**) Schematic representation of the injected and illuminated eye with the ROI used for measurements in AO images. (**D** and **D′**) AO images from the ROI selected from the injected eye (KR) and the injected and illuminated eye (KR + Illu) showing the PR distribution and segmentation in live animals at 6 months after illumination; (**E**) quantification of the dispersion, spacing, and density of PRs in the injected eye (KR, gray bars) and the injected and illuminated eye (KR + Illu, red bars). The KR controls (gray bars) are set to 100%, and the KR + Illu (red bars) are normalized against the controls. All values are shown as mean ± SD for *N* = 3 ROIs, *P < 0.05, **P < 0.001, and ***P < 0.0001 with unpaired Student’s *t* test. (**F**) Scotopic and photopic a- and b-wave amplitudes shown as a function of stimulus intensity (*x* axis) from NHPs before [baseline (BL), gray bars] and after illumination [3 months (3M), red bars]; all values are shown as mean ± SD for *N* = 4 NHPs.

We used the same construct of KR under the control of the hRho promoter and packaged it in AAV5, as this serotype has been shown to be most efficient in transducing PRs in NHPs (*41*). AAV5-h*Rho-KR* was delivered to both eyes of the NHP (cynomolgus *Macaca fascicularis*) by subretinal injections (Table 1). Eight weeks after injection, one eye was illuminated, while the other eye was left undilated and covered and served as a control. The live fundus autofluorescence image showed expression of KR starting from 4 weeks (Fig. 2A). KR immunolabeling on retinal flatmounts and sections revealed rod-specific expression with a distinct mosaic pattern observed in primate retinas, confirming that we were able to achieve expression only in rods and not in cones of NHP retina (Fig. 2, B and B′). After illumination, there is loss of these KR expressing rods (Fig. 2C). The progression of degeneration in live animals was monitored by OCT, adaptive optics (AO), and ERG. OCT imaging revealed areas of degeneration in the illuminated eye. The regions of disruption were localized to the ONL as seen in the area demarcated by the yellow dotted lines (Fig. 2, D and D″). Histological section of the KR-injected and illuminated eye revealed that there were broad regions where the ONL was thinner, whereas the adjacent regions showed intact ONL (Fig. 2E, regions E1 and E2). On the other hand, there were also regions where the disruptions were more punctuate, localized in smaller clusters showing disorganization of all layers of the retina (Fig. 2, E3 to E5). Rhodopsin immunolabeling revealed the loss of rod PRs and overall thinning of the ONL and retina in the KR-injected and illuminated eye compared to the KR-injected and nonilluminated control (Fig. 2, F to G′). Heatmaps were generated from the injected area after injection (KR) and 3 months after illumination (KR + Illu), and the thickness of the entire retina and the ONL was quantified. Three months after illumination, there is thinning of the retina (indicated by black arrows) (Fig. 3A). The thickness of the whole retina and the outer retina shows a slight reduction (Fig. 3B). AO imaging gave a clear indication of the degeneration. Small regions of interest (ROIs) were selected from the injected and noninjected parts of the retina of both the KR-injected but nonilluminated eye (KR-control) and the KR-injected and illuminated eye (KR + Illu) (Fig. 3C). Both the density and arrangement of the PRs were affected in the KR + Illu eye (Fig. 3, D and D′) with an 80% reduction in the density of PRs and a 27 and 54% increase in dispersion and spacing, respectively. On the other hand, the arrangement and density were not affected in the noninjected areas, although there was a significant effect on the dispersion showing a 38% increase (Fig. 3E). All the animals were housed in a 12-hour light/12-hour dark cycle (white spectrum containing green light in the 565 nm range). This results in some effect of the ambient light over a long time (8 months after injection) as seen in Fig. 3 (D and D′) (KR compared in the injected and outside the injected area). This can be further improved by changing light conditions in housing areas. The full-field ERG (ffERG) recorded under scotopic and photopic conditions revealed a general trend in reduction of the a- and b-wave amplitudes in the injected and illuminated eye, but this was not significant (Fig. 3F).

### Strategy 2: CRISPR-Cas9 strategy and proof of concept in rodents

**Fig. 4.**
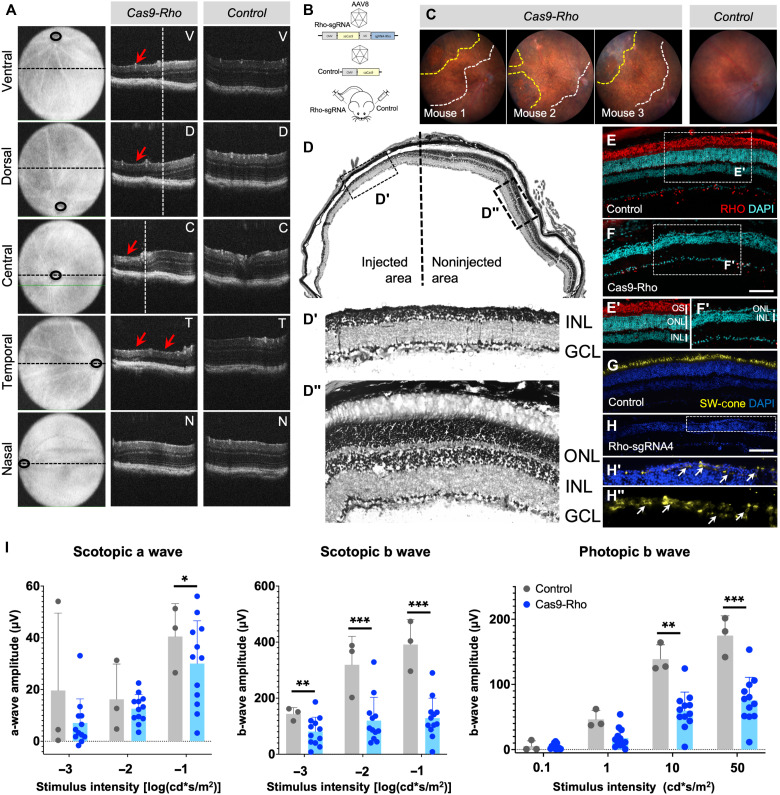
CRISPR-Cas9 strategy for rod ablation and proof of concept in mouse. (**A**) OCT images of mouse injected with Cas9 and *Rhodopsin* sgRNA and control mouse at the ventral (V), dorsal (D), central (C), temporal (T), and nasal (N) regions. Red arrows point to the regions of degeneration. (**B**) Schematic representation of the experiment protocol in mouse. Cas9 and guide RNA targeting *Rhodopsin* under the control of the CMV promoter is packaged into AAV vectors and delivered by subretinal injection to the retina. (**C**) Fundus images of three mice after injection. The white dotted line demarcates the injected area, and the yellow dotted line demarcates the area of damage. (**D**) Histology of the eye injected with Cas9-*Rho* showing the injected and noninjected region. Magnified images of the areas demarcated within the boxes are shown below—(D′) injected area and (D″) noninjected area. (**E** to **H″**) Immunolabeling of the (E and F) control eye and the eye injected with (F and H) Cas9-*Rho* with (E to F′) rhodopsin (in red) and (G to H″) short-wavelength (SW) cone opsin (G and H) (in yellow) and nuclear staining by DAPI (in cyan blue or blue). The box demarcated with the white dotted line in (H) is shown in (H′) and further magnified in (H″). White arrows point to cell bodies labeled with cone opsin. (**I**) Scotopic (a and b wave) and photopic b-wave amplitudes shown as a function of stimulus intensity (*x* axis) of control (gray bars) and Cas9-*Rho*–injected (blue bars) eyes. Values are shown as mean ± SD for *N* = 3 for control and *N* = 12 for Cas9-*Rho* eyes, and each group was compared by Student’s *t* test, *P < 0.05, **P < 0.001, and ***P < 0.0001. Scale bars, 100 μm.

For the next strategy, we used the CRISPR-Cas system that has proved to be an efficient tool for gene editing. The sequence comparison of the exon1 of *Rhodopsin* gene in mouse, macaque, and human showed high sequence similarity with some mismatches. Guide RNAs were selected using the Crispor software based on the predicted indel frequency and the off-targets. Four guide RNAs each specific to mouse, macaque, and human sequence were designed and cloned into an expression cassette with the Cas9 under the control of a cytomegalovirus (CMV) promoter (fig. S3A). The mouse-specific guides were tested in the PR cell line 661W, and the sgRNA4 generated the highest indels at 13% (fig. S3B). However, the transfection efficiency of this cell line was rather low (at 15%) (fig. S3B′). The macaque and human-specific sgRNAs did not result in any indels in 661W cells. Hence, we decided to test the macaque-specific sgRNAs in a monkey COS7 cell line and the human-specific sgRNAs in human embryonic kidney (HEK) cells. The macaque sgRNAs did not result in significant indels (fig. S3, C and C′). The human sgRNA2 at 7% indels was the best performing (fig. S3, D and D′). The human-sgRNA2 sequence was completely identical to the macaque-sgRNA1, and there was a 2–base pair (bp) difference from the mouse sgRNA4. Hence, we selected mouse sgRNA4 for the in vivo experiments in mouse and macaque sgRNA1 for NHP. OCT imaging revealed severe thinning of the outer retina restricted to the injected area 4 months after injection of sgRNA4 (Fig. 4A). The construct containing mouse sgRNA4 and CMV-Cas9 was packaged in AAV8 and delivered to the mouse outer retina by subretinal injections (Fig. 4B). Two months after injection, the degenerated areas were visible in the fundus images wherein the yellow dotted line demarcates the severely degenerated areas, while the white dotted lines show the injected area (Fig. 4C). This was further confirmed by imaging of histological sections that revealed complete loss of the ONL at 6 months after injection in the injected area (Fig. 4, D and D′), while the noninjected part of the same eye was intact (Fig. 4, D and D″). Immunolabeling of retinal sections with rhodopsin in the control area (Fig. 4, E and E′) and injected area (Fig. 4, F and F′) showed complete loss of rods in the injected area. Furthermore, short-wavelength cone-opsin labeling appeared to be absent in the injected area compared to the control (Fig. 4, G and H). The control and Cas9-*Rho* images were acquired with the same microscope settings and exposure. To further investigate whether dormant cones were present, the intensity of the signal was enhanced in the single layer of cell bodies. This enabled the visualizing of opsin labeling in the cell bodies, providing evidence for the presence of dormant cones after loss of rods (Fig. 4, H′ to H″, and fig. S5). Hence, we were able to achieve loss of PRs (both rods and cones) 6 months after Cas9-*Rho* injections. The loss of retinal function was tested by ERG by providing increasing flash intensities of light stimulus under scotopic and photopic conditions. There was a general trend in reduction of function in the Cas9-*Rho* group compared to the Cas9 control. The difference was more significant in the scotopic and photopic b-wave amplitudes.

### Strategy 2: CRISPR-Cas9–mediated RD in NHP

**Fig. 5.**
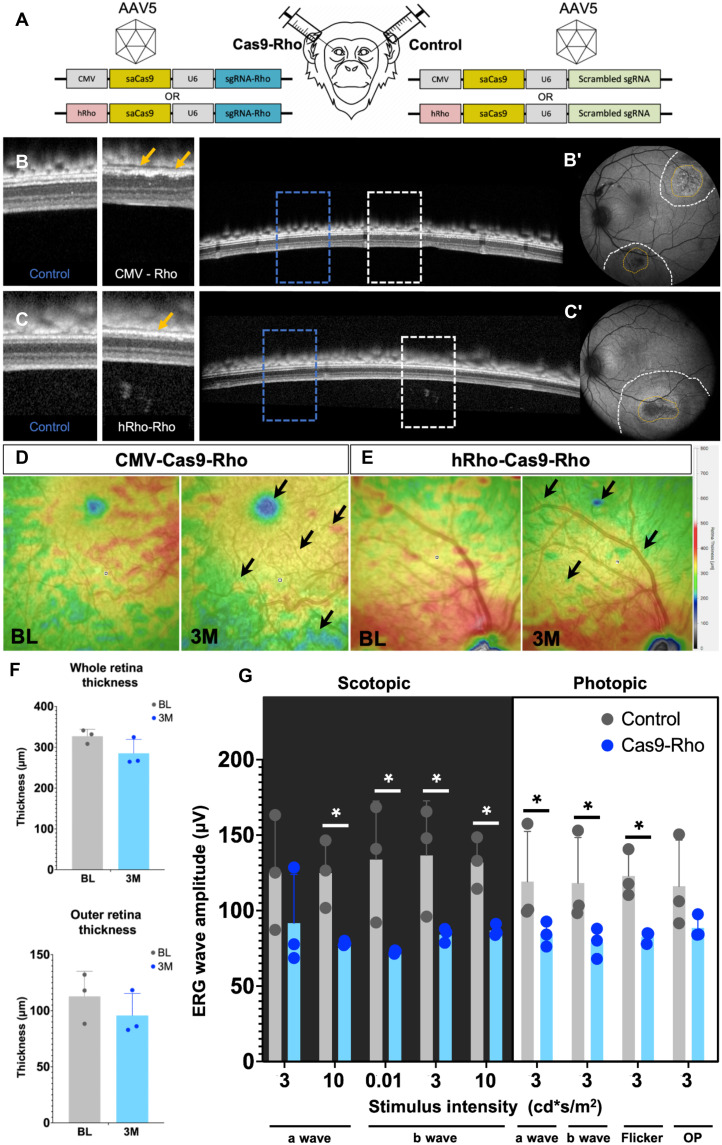
CRISPR-Cas9 loss of structure and function of NHP retina. (**A**) Schematic representation of the injection in NHP. Cas9 and guide RNA targeting *Rhodopsin* under the control of CMV or rhodopsin promoter is packaged into AAV vectors and delivered by subretinal injection to the retina. (**B** to **C′**) OCT images of NHP eyes injected with Cas9-*Rho* under the control of (B and B′) a ubiquitous (CMV) or a (C and C′) rod-specific (rhodopsin) promoter. The area demarcated by the dotted line and the corresponding magnified images show the unaffected regions (blue boxes as control) and the degenerated areas (white boxes). (B′ and C′) The injection bleb (white dotted line) and the focal regions of degeneration (yellow dotted line) are demarcated on the eye fundus image. (**D** and **E**) Heatmaps from the (D) CMV-Cas9-*Rho*–injected eye and (E) Rho-Cas9-*Rho*–injected eye at baseline before injection (BL) and 3 months after injection (3M). (**F**) Thickness of the whole retina and the outer retina 3M after injection. (**G**) Scotopic (a and b waves), photopic (a and b waves), flicker ERG, and oscillatory potential (OP) amplitudes shown as a function of stimulus intensity (*x* axis) from control eyes injected with Cas9-scrambled-sgRNA (BL, gray bars) and Cas9-*Rho* (blue bars); all values are shown as mean ± SD for *N* = 3 NHPs, and each group was compared by Student’s *t* test, *P < 0.05.

**Fig. 6.**
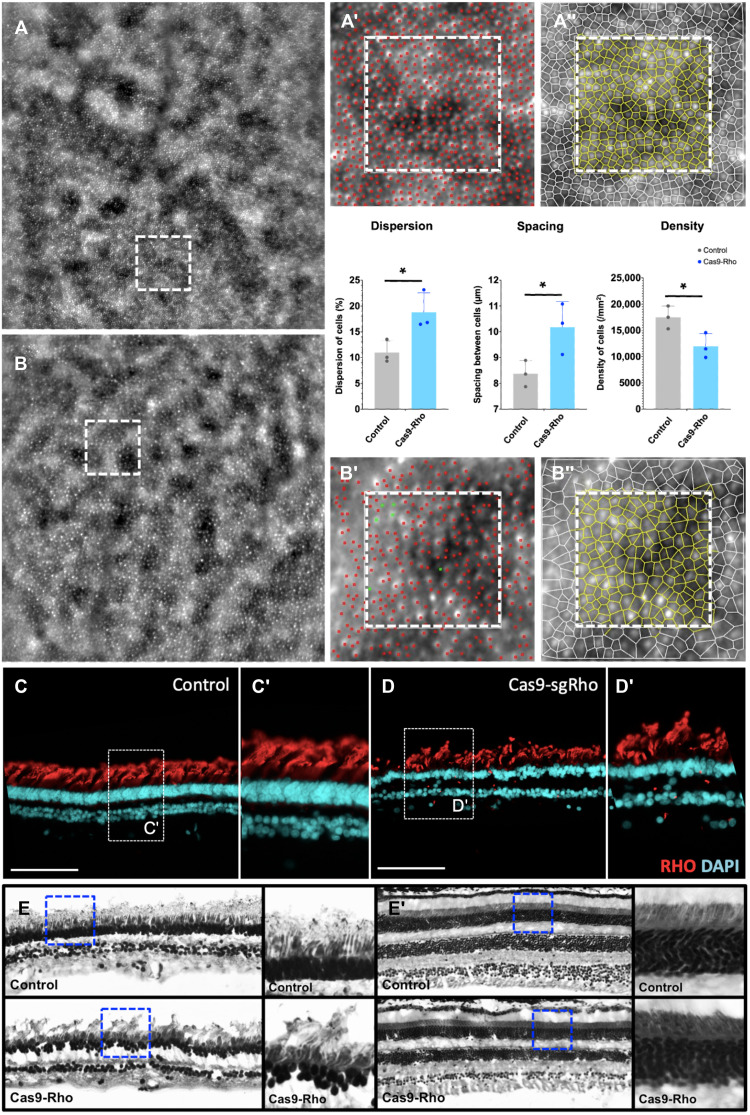
CRISPR-Cas9–mediated retinal degeneration in NHP. (**A** to **B″**) Adaptive optics images from the (A and B) ROI selected from the (A to A″) control eye and (B to B″) Cas9-*Rho* eye showing the PRs (A′ and B′) distribution and (A″ and B″) segmentation in live animals at 3 months after injection. Dispersion, spacing, and density of PRs are quantified in the control eye (gray bars) and Cas9-*Rho*–injected eye (blue bars). All values are shown as mean ± SD for *N* = 3 ROIs, *P < 0.05 with unpaired Student’s *t* test. (**C** to **D′**) Immunolabeling of rhodopsin in (C and C′) Control and (D and D′) Cas9-*Rho* eyes from the area of injection. (**E** and **E′**) Histological sections of the control and Cas9-*Rho* eyes from the (E) injected area and (E′) a distal region (E′). ROIs are demarcated within blue dotted lines and magnified in adjacent panels. Scale bars, 100 μm.

For NHP, we developed constructs containing the macaque-specific sgRNA1 targeting the exon1 of *Rhodopsin* gene under the ubiquitous CMV promoter (as used in the CRISPR proof-of-concept experiments in mice) or under a rod cell–specific promoter hRho (as the one used in the *KillerRed* experiments). For the control eye, a Cas9-scrambled guide was used under the control of CMV or hRho promoters. These constructs were packaged into AAV5 vectors and delivered to the adult NHP eyes by subretinal injections (Fig. 5A) (Table 1). The OCT images 3 months after injection revealed areas of degeneration that were restricted to the outer retinal layers and more specifically to the PR segments. These ONL disruptions were more severe in the NHP that received Cas9 expressed under a ubiquitous promoter compared to the one that received it with rod cell–specific promoter–hRho (Fig. 5, B to C′). Heatmaps generated from the injected areas of the control and Cas9-*Rho* eyes 3 months after injection revealed a thinning of the retina (indicated by black arrows) (Fig. 5, D and E). The thickness of the whole retina and the outer retina was quantified from three NHPs and showed a slight reduction 3 months after injection (Fig. 5F). FfERG recordings under scotopic and photopic conditions from the three NHPs showed a significant reduction in both the a- and b-wave amplitudes of the Cas9-*Rho* group (Fig. 5G). AO images from the injected area of the control eye and the Cas9-*Rho* eye were acquired for analysis and quantification. Both density and arrangement of the PRs were affected in the Cas9-Rho eye compared to the control. The density of PRs was 32% lower, while the dispersion was 42% higher and spacing was 18% higher compared to the controls (Fig. 6, A and B″). Rhodopsin immunolabeling revealed the loss of rod PRs and overall thinning of the ONL and retina (Fig. 6, C to D′). Reduction in immunolabeling of long-wavelength cone opsin was also observed (fig. S5, A to B′). However, the PR layer was not completely lost as seen in mice. Histological section of the Cas9-*Rho* injected eye revealed that there were broad regions where the ONL was thinner, whereas the adjacent regions showed intact ONL (Fig. 6, E and E′).

### Strategy 3: Physical barrier strategy and proof of concept in rodents

**Fig. 7.**
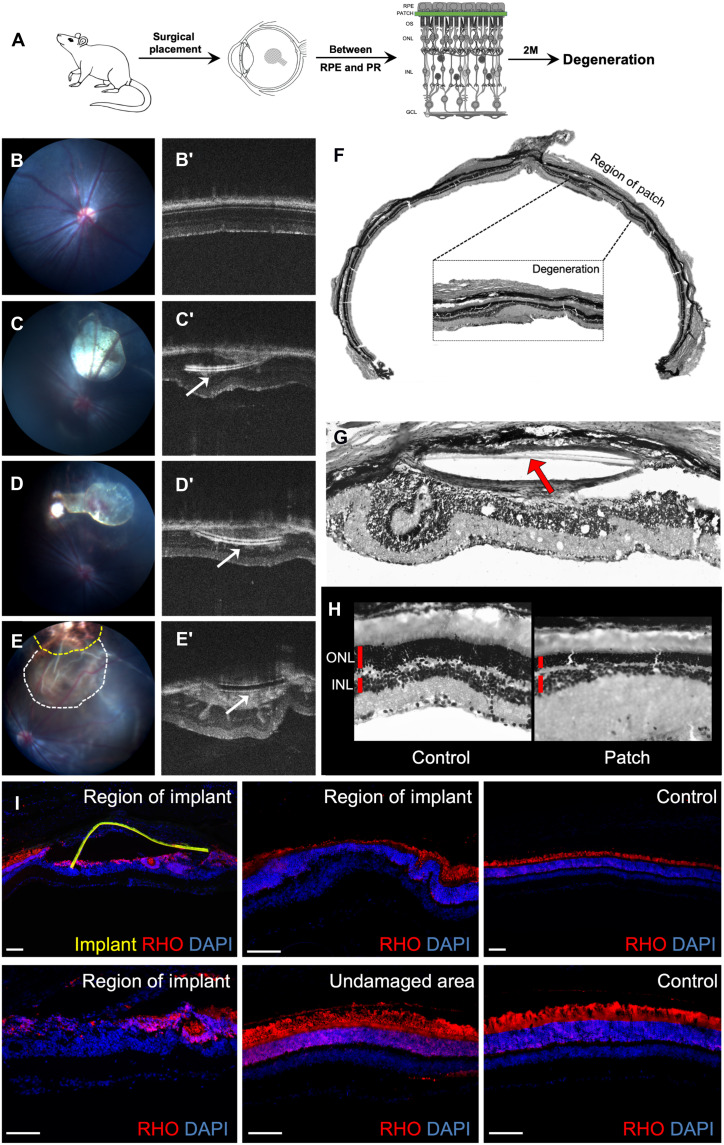
Physical barrier strategy and proof of concept in rat. (**A**) Schematic representation of the experiment protocol—a polymer patch is surgically placed between the RPE and the PRs of rat retinas. (**B** to **E′**) Fundus (B to E) and OCT images (B′ to E′) acquired before surgery (B and B′) and 4 weeks after surgery (C′ to E′) with polyimide patch (C and C′), parylene-coated polyimide patch (C and C′), and SU8 patch (E and E′); arrow points to the patch. (**F** and **G**) Histology of the eye with the red arrow pointing toward the polymer patch. (**H**) Magnified images of an area distal from the patch (control) and the area proximal to the patch (Patch). (**I**) Immunolabeling of different regions with rhodopsin (in red) and nuclear staining by DAPI (in blue). Undamaged area: distal region from the patch; control: contralateral eye without a patch. Scale bars, 100 μm.

To have a faster and acute model of degeneration, we envisioned introducing a physical barrier between the RPE and the PRs (PRs). Since the PRs depend on the RPE for their normal function and homeostasis, this loss of connection is likely to cause PR stress and eventual death. The proof-of-concept studies were carried out in rats as the mouse retina proved too small for such surgeries (Fig. 7A). The patches were made from three different materials—polyimide, polyimide coated with parylene, and SU8—and surgically placed between the RPE and PRs (Fig. 7, B to E′). The parylene coating provides a protective layer and allows long-term usage in contact with biological tissues. Both the polyimide (Fig. 7, C and C′) and polyimide + parylene (Fig. 7, D and D′) patches were comparable in terms of ease of placement and the level of degeneration achieved. Ideally, these patches will be placed for a specific duration of time, and when the desired level of PR loss is achieved, that patch will be removed to introduce a therapeutic agent (such as PR transplants) in the degenerated area. In this context, it would be desirable to have a patch that is biodegradable with which we could bypass the patch removal step. The SU8 patches were chosen for their biodegradable properties. However, 2 weeks after surgery, the patches already degraded and caused massive physical damage and inflammation in the surgery location and surrounding areas precluding the use of this material (Fig. 7, E and E′). Hence, polyimide + parylene patches were selected for further experiments in NHPs. The polyimide + parylene patch caused loss of ONL in the region of patch (Fig. 7, F and G) and overall thinning of the ONL in regions proximal to the patch when compared to control areas distal to the patch (Fig. 7H). Immunolabeling with rhodopsin revealed loss of PRs at the region of the patch compared to the undamaged area distal to the patch of the same eye or the contralateral control eye (Fig. 7I). Because of the small size of the eye, the patch causes a lot of physical damage during placement (Fig. 7, G and I). Reducing the dimensions (1.5 mm width with 15 μm thickness) further would make them very fragile and difficult to handle. Hence, further characterization of these patches in rats was not performed.

### Strategy 3: Physical barrier–mediated RD in NHP

**Fig. 8.**
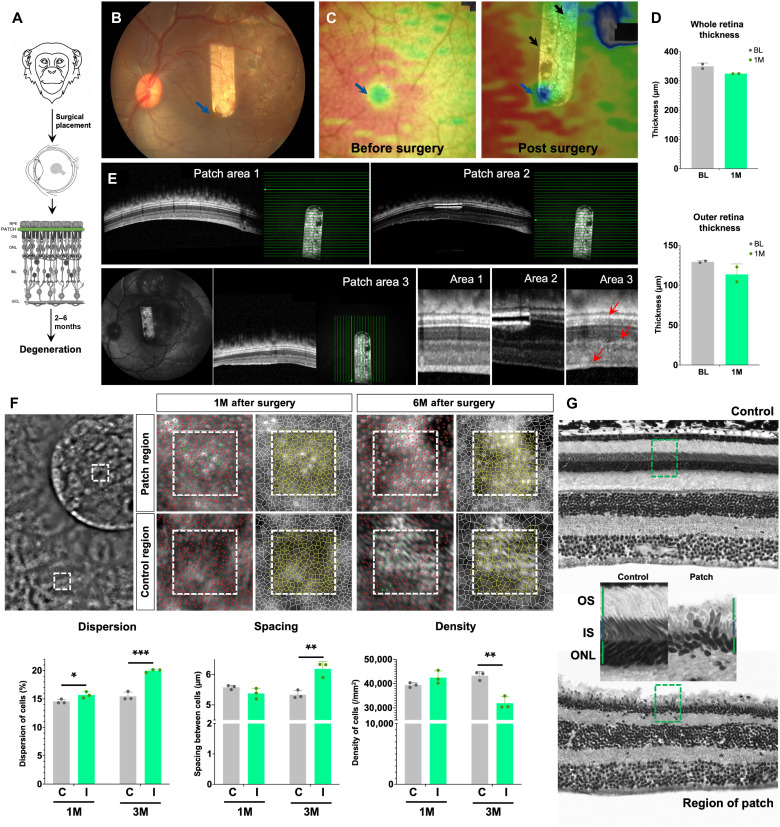
Physical barrier–mediated retinal degeneration in NHP. (**A**) Schematic representation of the experiment protocol—a polymer patch is surgically placed between the RPE and the PRs of the NHP retina. (**B**) Fundus image of the NHP retina showing the placement of the polymer patch in proximity of the fovea. (**C**) Heatmaps from the area of eye with the polymer patch before surgery and 1 month after surgery; black arrows point toward regions of degeneration and thinner areas of the retina, and blue arrow points to the fovea. (**D** and **D′**) Thickness of the (D) whole retina and the (D′) outer retina 1 month (1M) after injection. (**E**) OCT images from a region distal from patch (patch area 1), showing cross section of the patch (patch area 2) and in close proximity of the patch (patch area 3). The bright green lines show the exact region of the OCT images, and magnified images from each area are shown in the panels on the right. Red arrows point to punctuate retinal disorganization. (**F**) Adaptive optics image from the eye with patch showing the ROI in an area within the patch and control region as white boxes. The PR distribution and segmentation from the patch and control ROI are shown 1M and 6M after surgery. Dispersion, spacing, and density of PRs are quantified in the control region (gray bars) and patch ROI (green bars) at 1M and 6M after surgery. All values are shown as mean ± SD for *N* = 3 ROIs, *P < 0.05, **P < 0.001, and ***P < 0.0001 with unpaired Student’s *t* test. (**G**) Histological sections of the control eye and the eye with patch. ROIs are demarcated by the green dotted lines and shown magnified.

**Fig. 9.**
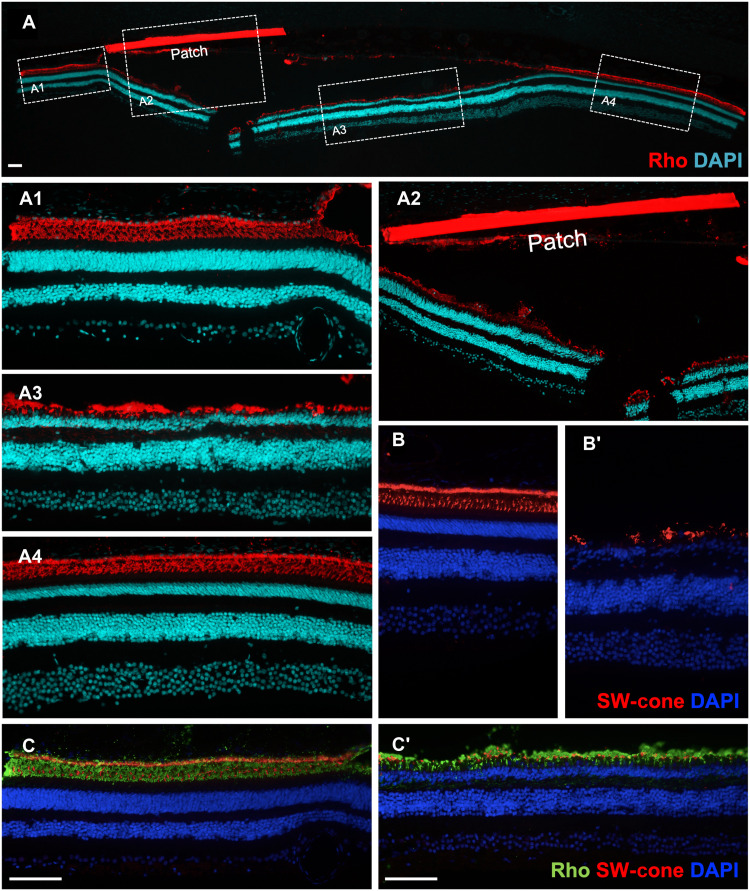
Rod and cone loss in NHP retina induced by physical strategy. (**A**) Immunolabeling with rhodopsin (in red) and nuclear staining with DAPI (in cyan blue) of the eye with the patch. The different ROIs are demarcated within white boxes and magnified in (A1) to (A4); (A1) region proximal to the patch, (A2) area with the patch, (A3) ONL just below the patch, and (A4) region distal to the patch. (**B** and **B′**) Immunolabeling with short-wavelength cone opsin (in red) and nuclear staining with DAPI (in blue) of the eye with the patch in a (B) proximal control area near the region in A4 and (B′) patch area near the region in A3. (**C** and **C′**) Coimmunolabeling with rhodopsin (in green), short-wavelength cone opsin (in red), and nuclear staining with DAPI (in blue) of the eye with the patch in a (B) proximal control area near the region in A1 and (B′) patch area near the region in A3. Scale bars, 100 μm.

The polyimide + parylene patch was placed surgically between the RPE and PR layer, and degeneration was analyzed between 1 and 6 months after surgery (Fig. 8A)(Table 1). The patch was placed close to the fovea (blue arrow) (Fig. 8B). One month after surgery, the thickness heatmaps showed thinning of the retina close to the patch region and the macular region (indicated by arrows) (Fig. 8C). Quantification of thickness revealed that at 1 month after surgery, the retina, particularly the outer retina, showed some thinning (Fig. 8D). The OCT images acquired 1 month after surgery showed that the regions distal to the patch (area 1) are unaffected, whereas the ONL was thinner in the region of the patch (area 2), and the retinal layers were slightly disorganized in the proximal regions (red arrows) (area 3) (Fig. 8E). AO images from the region with patch and a control region were acquired and quantified at 1 and 6 months after surgery. The density of PRs did not change at 1 month, but the arrangement was slightly affected as seen by a 1% increase in the dispersion. However, 6 months after surgery, there was a 26% reduction in the density of PRs, 23% increase in the dispersion, and a 14% increase in spacing (Fig. 8F). Comparison of the histological sections showed intact retinal layers and PRs in the control region, while there was loss of PRs and thinning of the retina in proximity of the patch (Fig. 8G). Immunolabeling with rhodopsin revealed thinner ONL in the region of the patch, while the adjacent areas remained intact. In the region of the patch, the ONL is thinner but not completely lost. However, disorganization of the outer retinal layers was apparent (Fig. 9, A to A4). This technique does not specifically target rods, so cone loss is also observed in the region of the patch (Fig. 9, B to C′, and fig. S5, C to D′).

## DISCUSSION

The field of ocular gene and cell therapies is experiencing a massive increase in the development and translation of experimental therapies into clinical application (*42*, *43*). However, there is an unmet need for relevant preclinical models that can be used for testing these therapies and understanding disease mechanisms. The several available rodent models serve well for proof-of-concept studies but lack comparable size and anatomical features (macula and fovea) that affect the manifestation and progression of the disease features (*3*). Here, we successfully generated three distinct animal models that can be used for testing different gene and cell-based therapies and understanding the progression of disease.

Our main aim was to generate a model for rod-cone dystrophy, which is primarily caused by mutations in rod PR genes (*44*). One of the important features of rod-cone dystrophies is that the rods are lost first, followed by cone loss because the cones depend on rods for survival factors (*45*–*47*). The AO imaging modality was particularly important to test this as it quantifies the cone PRs. In addition, in each of our models, about 3 to 6 months after induction of degeneration, we observe a reduction in the density of the cones, with disruptions in their spatial distribution. The “KillerRed model” mimics this by causing rod cell death by oxidative stress, and a reduction in cones can be observed 8 to 10 months after induction similar to the sequential events that occur in patients with rod-cone dystrophy. The “CRISPR-Cas9 model” is genetically representative of a common human rod-cone dystrophy as with this technique we specifically ablate a frequently mutated gene in rods by editing the *Rhodopsin* gene similar to the *RHO* mutation in human disease. With this strategy, reduction in the number of cones is observed 3 months after injection. In the “patch model,” we do not specifically target the rods but a small area of the retina with the intention of clearing both rods and cones in the region. This resulted in a reduction in cones after 6 months. Here, the cones are not lost as a consequence of rod death, but cones and rods are lost simultaneously. In the KR model, there is an increase in the dispersion of cones outside the injected area in the macular region, which is similar to the dispersion observed in the patch model at 1 month after surgery. This suggests that there is some lateral effect outside the injected area and probably some cell death and cone loss will be observed in the macular and foveal region with time.

The KR model allows spatial control as the degeneration will be restricted to the injected area, as well as only rods will be lost specifically because of the use of a rod-specific promoter. It further permits temporal control as the damage will be initiated only after exposure to light. Because of this unique feature, many macaques can be injected with KR, and when required for testing, the eye can be exposed to light. For light exposure, we used an LED light at approximately 565 nm at a specified distance from the eye, specific intensity, and exposure time. This can be further optimized to achieve better temporal control by modulating the duration of exposure and/or changing the distance, intensity, and type of LED used to cause focal or dispersed degeneration (depending on the requirement).

In the CRISPR model, we made an important observation for in vitro testing of guides. While designing and testing the guides for the CRISPR model, we used the retinal cell line 661W that was derived from retinal tumors of transgenic mice (*48*). We did not achieve a high transfection efficiency of this line (approximately 15%), but the guides designed for mouse version of *Rhodopsin* resulted in indels (the highest was nearly 12%). As expected, when we tested the macaque guides in a monkey cell line (COS7) and the human guides in a human cell line (HEK), we were able to generate indels before in vivo testing.

Degeneration of the retina occurs in stages, and the chosen vision restoration method largely depends on the type and stage of the disease. In the earlier stages, when the retinal cells are still present, neuroprotective factors can be provided to slow down the progression of the disease, and gene replacement or editing can be used to compensate the effect of gene mutations. On the other hand, in later stages, when the retinal cells are mostly lost, cell replacement therapy and placement of electrical implants could help in restoring some useful vision. In addition, specific therapies can control symptoms that arise from perturbed cellular mechanisms such as oxidative stress, inflammation, or neovascularization. Our models can be used for testing a wide range of these therapeutic strategies. There are many neuroprotective factors that have been tested in rodents and shown to slow down or prevent degeneration such as bile acids, dopamine, steroid hormones, and neurotrophic factors (glial-derived neutotrophic factor, brain-derived neurotrophic factor, ciliary nerve trophic factor, nerve growth factor, etc.) (*49*). All three models can be used for testing such factors. One potent factor known as RdCVF (rod-derived cone viability factor) is secreted by rods and has been shown to be responsible for the eventual cone death after rod loss (*46*, *50*). The neuroprotective effect of RdCVF can be tested in the KR and CRISPR models, because these models specifically cause rod death, and by AO, we were able to show that the cone death follows. The three models can also be used for testing specific gene therapies provided the cell type where the gene is to be expressed is still present. However, repeat injections to the same area can be technically challenging and undesirable because of the possibility of surgical damage and potential inflammation caused by anti-AAV immune responses (*51*, *52*). To circumvent these novel AAV variants, nonviral vectors or intravitreal modes of delivery may be considered.

All three models can be used for testing mutation-independent therapies for late-stage degeneration such as cell transplantation and artificial retinal stimulation by electrical prosthesis or optogenetic therapies. Generation of PRs from induced pluripotent stem cells is well established now (*53*, *54*). However, attempts to transplant these cells into rodents require strong immunosuppression to avoid graft rejection of human cells in rodents. This needs to be taken into account while designing experiments alongside other factors such as material transfer and lack of proper integration (*55*, *56*). However, how the transplanted cells will behave in NHP is not known, and the models developed here can be useful to examine the effects of this intervention. The recent clinical success with a patient who had his vision restored following optogenetic therapy (*57*) is likely to increase the need for preclinical testing of such therapies. Such therapies can be tested in both the CRISPR model and the patch model by expressing the optogene either in the remaining dormant cones or other cell types of the retina (such as ganglion cells and bipolar cells). The KR model can also be considered for such therapies as well as for tests of retinal prosthesis. However, the retinas response to two surgeries (placing the patch and then replacing it by a retinal implant) is still to be tested.

Although rod-cone dystrophies are inherited conditions, nongenetic factors such as oxidative stress and inflammation are important common mechanisms involved in their pathogenesis and progression (*58*). The retina is a metabolically demanding tissue with a high oxygen consumption rate. In addition, the opsin molecules inside the PRs are being constantly subjected to light and oxidative stress, which further results in accumulation of ROS in RPE. Oxidative stress can result in activation of specific genes, transcription factors, or microRNAs that alter key pathways to further alter cellular response such as microglia activation, gliosis, and cell death (*59*). In our KR model, light-induced activation of KR causes degeneration by oxidative stress, and hence, this model provides an opportunity to study molecular mechanisms involved in oxidative stress associated with RD.

In conclusion, we have addressed an important unmet need in the field and propose three preclinical models that cause degeneration by distinct mechanisms following different timelines corresponding to the various needs of translational researchers working in the field of retinal disease. We have used multiple imaging modalities to monitor the progression of the degeneration in live animals, which can be further used to monitor the improvement after therapy. Obtaining significant data with NHP studies implies high cost, time, and effort, and by investing these in our models, we have secured robust and relevant data that will make these models suitable for testing various mutation-dependent and -independent therapies.

## MATERIALS AND METHODS

### Animals and ethics statement

WT C57BL/6J mice and Long-Evans rats were acquired from Janvier Labs, and the NHPs (cynomolgus *M. fascicularis*) were acquired from Noveprim, Mauritius. All mice and rats were housed under a 12-hour light/12-hour dark cycle with food and water ad libitum. The NHPs were screened for pre-existing antibodies against AAV5, and only seronegative animals were used in the study. The NHPs were regularly assessed by clinical observation, monitoring of food consumption and body weight, and ophthalmic examination after injection (fundus imaging, slit-lamp examination, OCT, and AO). All animal experiments and procedures were ethically approved by the French “Ministère de l’Education, de l’Enseignement Supérieur et de la Recherche” and were carried out according to institutional guidelines in adherence with the National Institutes of Health (NIH) *Guide for the Care and Use of Laboratory Animals* as well as the Directive 2010/63/EU of the European Parliament.

### Plasmids and AAV production

The KR from the plasmid vector p*KillerRed*-mem (Evrogen, Moscow, Russia) was cloned with a human rhodopsin promoter. The resulting plasmid hRho-KR was packaged into the AAV8 for injections in mice and into AAV5 for injections in NHPs. On the basis of the *Rhodopsin* gene sequence of *Mus musculus*, *M. fascicularis*, and *Homo sapiens*, we identified all Protospacer Adjacent Motif (PAM) sequences for SaCas9 on the exon1 of the *Rhodopsin* gene. For each species, the top four sgRNA sequences were selected on the basis of the high on-target score and low off-target score predicted by CRISPOR (http://crispor.tefor.net/). To construct SaCas9 and sgRNA-expressing plasmid, the selected sgRNAs were subcloned into pX601-AAV-CMV::NLS-SaCas9-NLS-3xHA-bGHpA;U6::Bsa I–sgRNA plasmid (pX601) (Addgene, plasmid 61591 from F. Zhang). The Cas9 and Cas9-Rho guide–containing plasmids were packaged into AAV8 (for injections in mice) and into AAV5 (for NHP injections). Recombinant AAVs were produced using the triple-transfection method on HEK293 cells [American Type Culture Collection (ATCC), CRL-1573], harvested 24 to 72 hours after transfection and purified by iodixanol gradient ultracentrifugation (*60*). The 40% iodixanol fraction was collected after a 90 min spin at 354,000*g*. Concentration and buffer exchange were performed against phosphate-buffered saline (PBS) containing 0.001% Pluronic. AAV vector stock titers were then determined on the basis of real-time quantitative polymerase chain reaction (PCR) titration method using Inverted Terminal Repeats (ITR) primers (*61*) and SYBR Green (Thermo Fisher Scientific).

### Cell transfection and indel analysis

661W cells (RRID: CVCL_6240), NIH 3T3 (ATCC, CRL-1658), COS-7 cells (ATCC, CRL-1651), and HEK293T cells (ATCC, CRL-3216) were grown in a 75 cm^2^ flask with a culture medium composed of Dulbecco’s modified Eagle medium (DMEM), high glucose (Gibco) supplemented with 10% (v/v) of heat-inactivated fetal bovine serum (Gibco) and 1% (v/v) of penicillin/streptomycin (Gibco) in an incubator at 37°C with a 95% O_2_, 5% CO_2_ humidified atmosphere. Cells were passaged using trypsin-EDTA 0.05% (Gibco) every 3 or 4 days at a split ratio of 1:20. Twenty-four hours before the transfection, 8 × 10^4^ 661W cells were seeded in a 24-well culture plate with DMEM and transfected with the plasmids containing guides using Lipofectamine cells that were harvested 72 hours following the transfection, and genomic DNA was then extracted using a NucleoSpin Tissue kit (Macherey-Nagel) following the manufacturer’s recommendation.

For indel analysis, primers were designed to amplify a ~700 bp genomic regions flanking the sgRNA target site with primers annealing at least 200 bp upstream or downstream the cleavage site. PCR products were then purified using NucleoSpin Gel and PCR Clean-up and sent for Sanger sequencing (Eurofins Genomics) using the same forward and reverse primers. Indel values were obtained using the Tracking of Indels by Decomposition (TIDE) web tool (http://shinyapps.datacurators.nl/tide/) as described previously (*62*).

### Subretinal injections

WT C57BL/6J mice (Janvier Labs) were used in this study and injected at 6 weeks of age. The mice were anesthetized by isoflurane inhalation. Following dilation of the pupils, 1 μl of the AAV was delivered subretinally using a 33-gauge needle. In KillerRed mice, the dose injected was 1 × 10^10^ vg. The CRISPR-Cas9 was injected at a dose of 1 × 10^9^ or 5 × 10^9^ vg.

Cynomolgus macaques (Noveprim, Mauritius) were anesthetized with an intramuscular injection of ketamine (10 mg/kg; Imalgene 1000, Merial) and xylazine (0.5 mg/kg; Rompun 2%, Bayer). Anesthesia was maintained with an intravenous injection of propofol (1 ml/kg per hour) [PropoVet Multidose (10 mg/ml), Zoetis]. Their pupils were dilated, and their eyelids were kept open using eyelid speculum. To perform subretinal AAV injections, two 25-gauge vitrectomy ports were set approximately 2 mm posterior to the limbus, one for the endoillumination probe and the other for the subretinal cannula. A 1 ml Hamilton syringe equipped with a 25-gauge subretinal cannula with a 41-gauge tip was used for the injection. The endoillumination probe and cannula were introduced into the eye. The viral vector solution (100 μl) was injected subretinally to create a bleb on the dorsal side of the fovea. The instruments were then withdrawn. Postinjection eyes received the ointment Fradexam (Labortoire TVM, France) consisting of a corticosteroid (dexamethasone disodium phosphate, 0.76 mg) and an antibiotic (framycetin sulfate, 3150 UI). In case of appearance of any inflammatory signs following the viral injection, the eyes were treated by periocular injection of 12 to 20 mg of Kenacort (Bristol-Myers Squibb) in addition to the ointments. In case this treatment was insufficient, systemic (intravenous and/or intramuscular) injections of corticosteroids were provided.

### In vivo imaging (fundus and OCT)

Mice pupils were dilated using 0.5% tropicamide (Mydriaticum, Théa Pharmaceauticals, France) and 5% phenylephrine (Néosynepherine, Europhta, France). The animals were anesthetized by isoflurane (Axience, France) inhalation, and the eyes were kept moistened with Lubrithal (Dechra Pharmaceuticals) during the course of the experiment. Fundi were monitored and photographed in two modalities (bright-field and red fluorescence) using the Micron IV mouse imaging system (Phoenix Research Laboratories, Pleasanton, CA, USA). For spectral-domain optical coherence tomography (SD-OCT), the mice were placed in front of the SD-OCT imaging device (Bioptigen 840 nm HHP; Bioptigen, NC, USA). The eyes were kept moisturized with 0.9% NaCl throughout the procedure. Image acquisition was done by using the rectangular scanning protocol consisting of 1000 A-scans (lines) and 100 B-scans (frames) with 16 frames/B-scan. The images were acquired in the dorsal, ventral, nasal, temporal, and central regions of the retina. The images acquired were processed with Fiji software (available at http://fiji.sc/Fiji).

For NHPs, the anesthesia and pupil dilation were carried out as described earlier. Spectralis High Resolution Angiography (HRA) + OCT system (Heidelberg Engineering, Germany) was used to acquire the fundus images and fluorescent images using the fundus autofluoresence mode (at an excitation wavelength of 488 nm and a barrier filter of 500 nm), as well as the B-scan SD-OCT acquisition.

### Adaptive optics

The rtx1 (Imagine Eyes, Orsay, France), a compact AO-Flood-Illumination Ophthalomoscope (FIO) commercially available device with a lateral resolution of 2 μm, was used to image the PR layer of the NHP. In the rtx1, retinal images are captured by a low-noise high-resolution charge-coupled device camera (Manta, Allied Vision, Stadtroda, Germany), while an AO correction system composed of a Shack-Hartmann wavefront sensor (HASO4 first, Imagine Optic, Orsay, France) and a deformable mirror (mirao52-e, Imagine Eyes) works in closed loop to compensate for the aberrations introduced by the eye. Retinal images obtained with the rtx1 have a resolution of 2 μm over a 4 × 4 degree field of view. A defocus of +0.50 diopters (corrected by a modified Badal assembly incorporated in the device) was used. NHPs were anesthetized during data acquisition because fixation could not be maintained otherwise. Anesthetized animals were laid on the examination table with their head fixed to have their eyes aligned with the camera. Because eyes cannot be manually moved during the experiments (otherwise, too much astigmatism is induced, avoiding proper imaging), the entire table needed to be positioned to explore intended retinal areas. The investigator (A.D.) followed the same procedure during each acquisition: (i) locating the fovea with the help of the retinal vascular pattern and then (ii) capturing at least five images centered and around the ROI site, as far as technically achievable. The focus (related to the axial depth in micrometers) was set between “0” and “+50” to capture the PR layer as sharp as possible. NHPs were dilated and anesthetized as mentioned above. The acquisition consisted of a series of 40 frames over 2 s with an exposure time of 10 ms (averaged by the embedded software to produce one final image) in a 4 × 4 degree field size, captured at each retinal location mentioned above. Then, the manufacturer commercial software (AODetect 3.0, Imagine Eyes, Orsay, France) was used for cone detection, segmentation, and regularity analyses. In a manually set sampling window of 80 × 80 pixels of each acquired retinal area (ROI), cones were first marked semi-automatically (automatic detection by the software, manually adjusted by E.B.), and the following estimates of density (Voronoi cell density), intercell spacing (the mean distance between each cone PR and its neighbors), and regularity index were obtained. The sampling window for ROI was placed at the closest location that was free from large vessels to avoid vascular artifacts.

### Electroretinogram

Mice were dark-adapted overnight and anesthetized with a mixture of ketamine (80 mg/kg; Imalgene 1000, Merial) and xylazine (8 mg/kg; Rompun 2%, Bayer). Their pupils were dilated as described before, and the cornea was anesthetized by local application of 0.4% oxybuprocaine (Théa Pharmaceauticals, France). Upper and lower lids were retracted to keep the eye open and proptosed. A contact lens–type electrode contacting the cornea through a layer of lubrithal gel (Dechra Pharmaceuticals) was used to record the retinal response, with needle electrodes placed on the head and back used as the reference and ground electrodes, respectively. Body temperature was maintained at ~37°C with a heating pad. The light stimulus was provided by a LED stimulator (ERG Espion from Diagnosys). Responses were amplified and filtered (1 Hz low and 300 Hz high cutoff filters) with a one-channel DC/AC amplifier. Dark-adapted (scotopic) responses were measured in darkness, during flash stimulations of 0.001, 0.01, and 0.1 cd·s/m^2^. Photopic ERGs were recorded at stimulations of 0.1,1, 10, and 50 cd·s/m^2^, following a 5-min light adaptation. Each dark-adapted or photopic ERG response is the mean of five responses from a set of five stimulatory flashes.

For NHPs, the anesthesia and pupil dilation were carried out as described earlier. The RETI-map-animal system (Roland Consult, Germany) was used to record full-field (ffERG) under scotopic and photopic conditions. The scotopic responses were measured after 20 min of dark adaptation during flash stimulations of 0.0095, 3.0, and 9.49 cd·s/m^2^. Photopic ERGs were recorded at stimulations of 3.0 cd·s/m^2^, following a 10-min light adaptation. The dark-adapted or photopic ERG responses are a mean of 20 to 24 responses.

### Illumination

Mice were anesthetized with a mixture of ketamine (80 mg/kg; Imalgene 1000, Merial) and xylazine (16 mg/kg; Rompun 2%, Bayer), and their pupils were dilated as described earlier. NHPs were anesthetized, and their pupils were dilated as described earlier, and their eyelids were kept open using an eyelid speculum. The animals were placed on a heating pad, and a coverslip was placed on each eye after application of lubrithal gel. The eyes were illuminated with green LED light (540 to 580 nm), directed toward the eye at a distance of 10 cm, so the eyes received ~5000 lux intensity of light. For mice, each eye was exposed to light for a total duration of 3 hours (in two sessions of 1.5 hours each, 1 week apart), and for NHPs, each eye was exposed for a total duration of 4 hours (in two sessions of 2 hours each, 1 week apart). After illumination, mouse eyes were treated with topical application of Ophthalon gel and the NHP eye with an ophthalmic Vitamin A Dulcis ointment (Allergan, Ireland). Whenever one eye of a mouse was used as nonilluminated control, the eye was not dilated and was covered with an opaque eye patch.

### Surgery for placement of patches

All rats were unilaterally implanted at 8 weeks of age. The surgery consisted of placing the patch in the subretinal space in the central region next to the optic nerve as previously described (*63*). Briefly, analgesia was provided with subcutaneous injection of buprenorphine (0.05 mg/kg) (Buprecare, Axience), and anesthesia was provided by 5% gaseous induction of isoflurane maintained at 2 to 3%. An additional corneal anesthesia was administered using oxybuprocaïne chlorohydrate eye drops. The eye was dilated by application of tropicamide (Mydriaticum 0.5%) solution. The animal was placed on a heating platform at 37°C to maintain body temperature throughout the procedure. A small sclerotomy was performed on the dorsal sclera tangential to the cornea. Sodium chondroitin sulfate–sodium hyaluronate (Viscoat Alcon) gel was injected in the sclerotomy to generate a retinal detachment. The implant was then inserted below the detached retina in the subretinal space in a location adjacent to the optic disk. At the end of surgery, the animal was placed in a recovery chamber at 30°C, and postoperative monitoring was conducted with postoperative analgesia.

NHPs were anesthetized by an intramuscular injection of ketamine (10 mg/kg) and xylazine (5 mg/kg) and maintained with an intravenous infusion of propofol (1 ml/kg per hour), followed by a local ocular anesthesia (oxybuprocaine chlorhydrate, Thea). Pupillary dilatation was achieved with 0.5% tropicamide eye drops (Mydriaticum) at least 20 to 25 min before intervention. The nonoperated eye was protected with an ophthalmic gel (Lubrithal, Dechra) to prevent dehydration. Subretinal implantation was performed using a three-port pars plana vitrectomy system with cold vitreous cavity irrigation (BBS Plus, Alcona) under a stereomicroscope (Lumera 700, Zeiss). The vitrectomy (23-gauge ports) was realized using Alcon Constellation. A bleb for the retinal detachment was then created by subretinal injection of BSS Plus with a subretinal cannula near the macular area. Before retinotomy, the appropriate area of the retina was coagulated. A retinotomy of 2 mm was performed using microscissors. The scleral incision of 2 mm was created to introduce the delivery system via the pars plana into the vitreous cavity. The patch was then delivered through the retinotomy, and the sclerotomy was finally closed. The retina was attached under perfluorocarbon liquid (DECA, DORC). A laser photocoagulation (Vitra Laser, Quantel Medical) was then applied to the area of the retinotomy. After a fluid/air followed by an air/20% SF6 gas (Physiol) exchange, the trocars were removed, and the additional portion of the 20% SF6 gas was added if needed. The conjunctiva was closed with an 8-0 suture, and the animal was allowed to awake progressively by intravenous propofol withdrawal.

### Tissue preparation, immunohistochemistry, and microscopy

To prepare retinal sections from mice and rats, the eyes were enucleated and fixed in 4% paraformaldehyde (PFA) prepared in PBS overnight. Cornea and lens were removed, and the eyecups were transferred into a tube containing 30% sucrose in PBS. For NHPs, the eye was fixed in 4% formaldehyde (Sigma-Aldrich) for 4 hours at room temperature (RT), followed by dissection and removal of cornea and lens. The eye cup was then transferred into PBS. A 50 mm section was cut out from the ROI and fixed overnight at 4°C. The dissected part was then treated with a sucrose gradient—10% for 1 hour, 20% for 1 hour, and 30% overnight at 4°C. Eyecups (for mice and rats) and dissected region (for NHP) were then embedded in tissue optimal cutting temperature compound (Microm Microtech, France) and snap frozen in liquid nitrogen. Sections (12 μm thick) were cut on a cryostat (Leica Biosystems), air dried, and stored at −80°C till further use. To prepare retinal flatmounts, mouse eyes were enucleated and incubated for 5 min in 2% PFA. After having removed the cornea and lens, the eyecup was cut in a cloverleaf shape by making four incisions. The retina and RPE were dissected from the sclera and placed separately in PBS in a 48-well plate. Dissected retinas and RPEs were postfixed at RT for 1 hour with 4% PFA. Similar procedure was followed to prepare flatmounts of the dissected region of the NHP retina.

The retinal sections were incubated with blocking solution (3% goat serum + 0.3% Triton X-100 in PBS) for 2 hours at RT (10% goat serum was used for blocking of the retinal and RPE flatmounts), washed with PBS, followed by overnight incubation with the primary antibody at 4°C. The primary antibodies used are KillerRed (Evrogen, AB961), rhodopsin (Sigma-Aldrich, MABN15), and short-wavelength cone opsin (Santa Cruz Biotechnology, sc14363). The following day, the tissue was washed with PBS and incubated with secondary antibody conjugated with Alexa Fluor 488 dye (emission in the green channel) or Alexa Fluor 594 dye (red channel) for 1 hour at RT. DAPI (4′-6′-diamino-2-phenylindole, dilactate; Invitrogen) was used for nuclear staining. After PBS washes, the retinal flatmounts or cryosections were mounted onto slides using Vectashield mounting medium (Vector Laboratories, USA) and protected with coverslips. Immunofluorescence was visualized using an Olympus upright confocal microscope and then analyzed with Fiji software.
