# Supplementary material for: Inducible nonhuman primate models of retinal degeneration for testing end-stage therapies
Source: Sci Adv. 2023 Aug 2;9(31):eadg8163. doi: 10.1126/sciadv.adg8163 (PMC10396314; doi:10.1126/sciadv.adg8163)
Supplement: Supplementary file 1 — Figs. S1 to S5 [file sciadv.adg8163_sm.pdf]

Science Advances
AAAS

Supplementary Materials for

Inducible nonhuman primate models of retinal degeneration for testing end-stage therapies

Divya Ail *et al.*

Corresponding author: Divya Ail, divya.ail@inserm.fr; Deniz Dalkara, deniz.dalkara@inserm.fr

Sci. Adv. **9**, eadg8163 (2023)
DOI: 10.1126/sciadv.adg8163

This PDF file includes:

Figs. S1 to S5

SUPPLEMENTARY MATERIALS

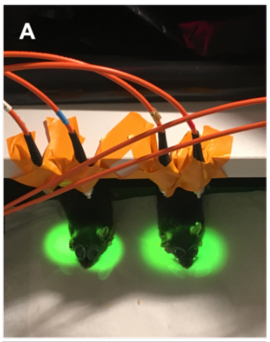

B

Level	Photon Flux (photon/ cm^2.s)	Illuminance (Lux)
6	1.00E+17	12485
4	3.83E+16	5445
2	6.40E+15	863

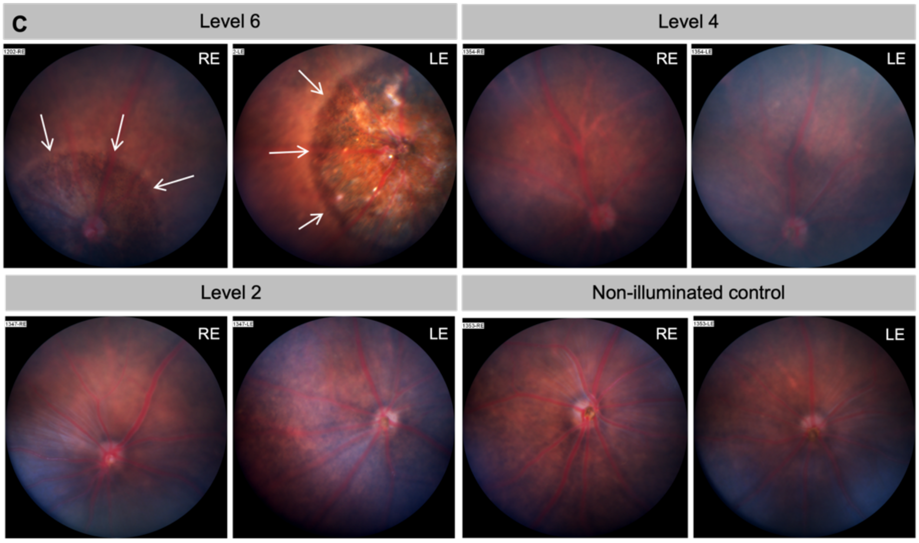

Supplementary figure S1. Optimization of the light intensity for KillerRed activation (A) Set-up of the 565nm LED lights used for activation of KillerRed; (B) Table showing the intensity levels in the apparatus and the corresponding values in Photon flux and luminance; (C) Exposure of mice eyes to Intensity levels 6, 4, 2 and non-illuminated controls. Arrows point to regions of damage. LE: Left eye, RE: Right eye.

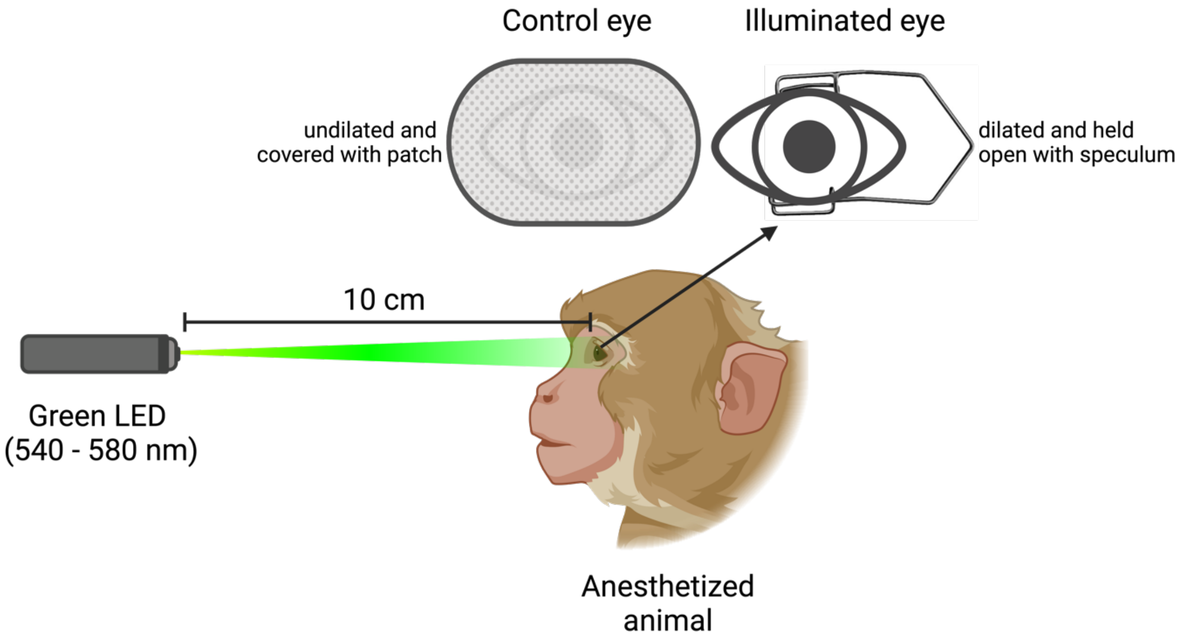

Supplementary figure S2. Illustration of illumination in nonhuman primate. Both eyes of the animal are injected with the same dose of AAV5-KillerRed. One eye is dilated and held open using a speculum (KR+Illumination), while the other eye (Control) is left undilated and covered with an eye-patch. A green LED light (approximately between 540 and 580nm) is placed at a distance of 10cm from the eye for 2 hours.

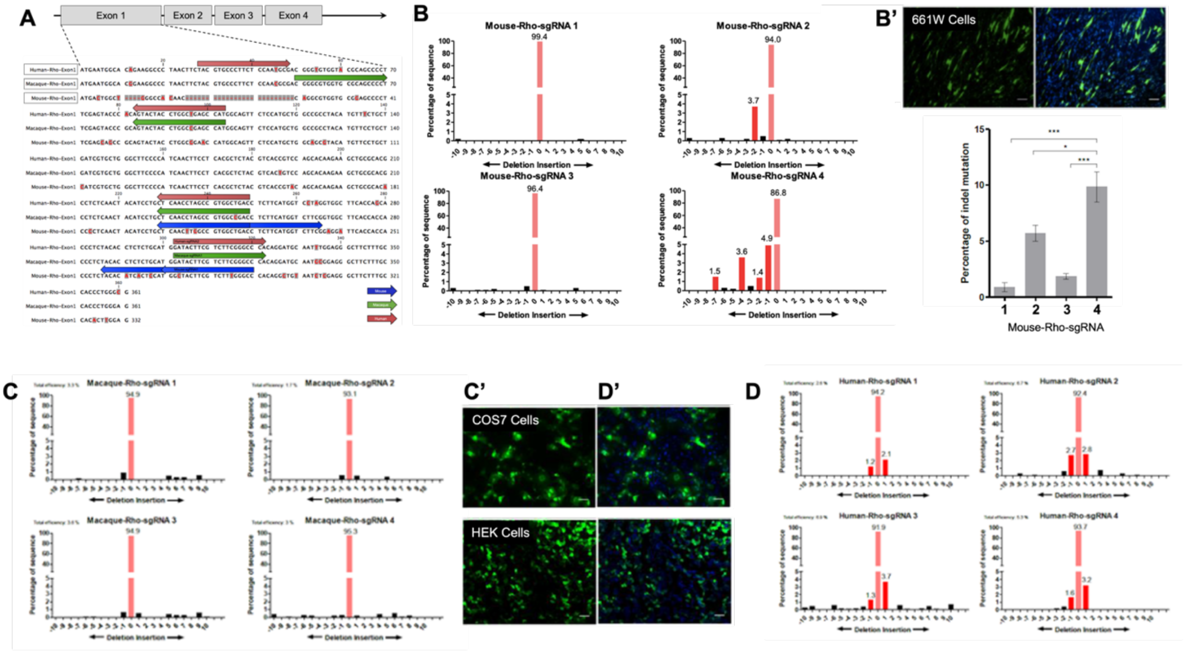

Supplementary figure S3. Design and selection of Rhodopsin specific guide RNAs (A) Schematic of the first 4 exons of the rhodopsin gene showing the sequence of exon1 in mouse, macaque and human. The bases that do not match are highlighted. The sites targeted by guide RNAs designed for mouse (in blue), macaque (in green) and human (in pink) are shown as arrows above the sequence; (B) Indel analysis of the 4 mouse-specific guides tested in the photoreceptor cell line-661W; (B') Transfection efficiency tested by GFP in 661W cells, (B") Comparison of the percentage of Indels caused by the 4 guides; (C) Indel analysis of the 4 macaque-specific guides tested in the COS7 cells; (C') Transfection efficiency tested by GFP in COS7 cells; (D) Indel analysis of the 4 human-specific guides tested in the HEK cells; (B') Transfection efficiency tested by GFP in HEK cells.

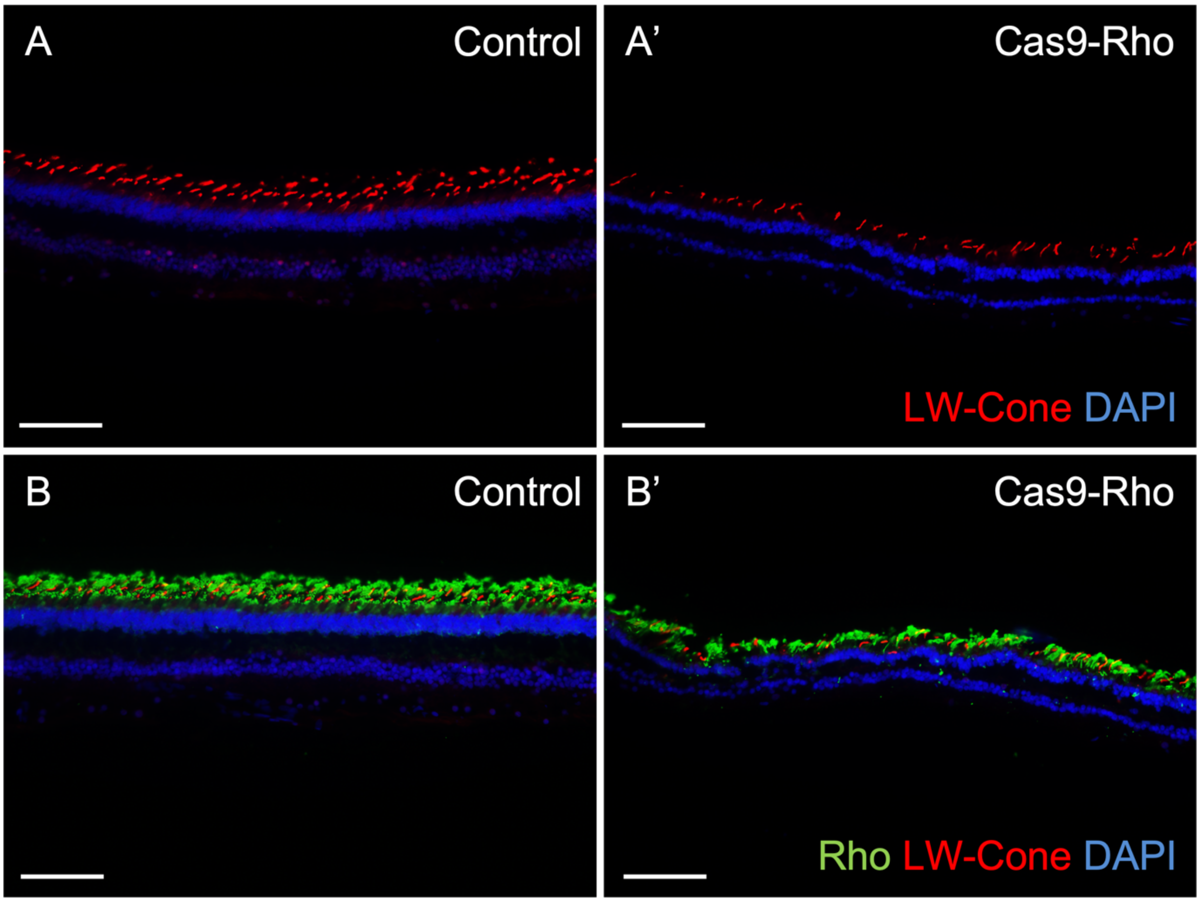

Supplementary figure S4. Immunolabeling of cones in CRISPR-strategy NHP model (A) Control and (A’) Cas9-Rho retinal sections immunolabelled with long-wavelength cone opsin (in red); (B) Control and (B’) Cas9-Rho retinal sections co-immunolabelled with rhodopsin (in green) and long-wavelength cone opsin (in red). Nuclear staining is done with DAPI (in blue). Scale bars indicate 100µM.

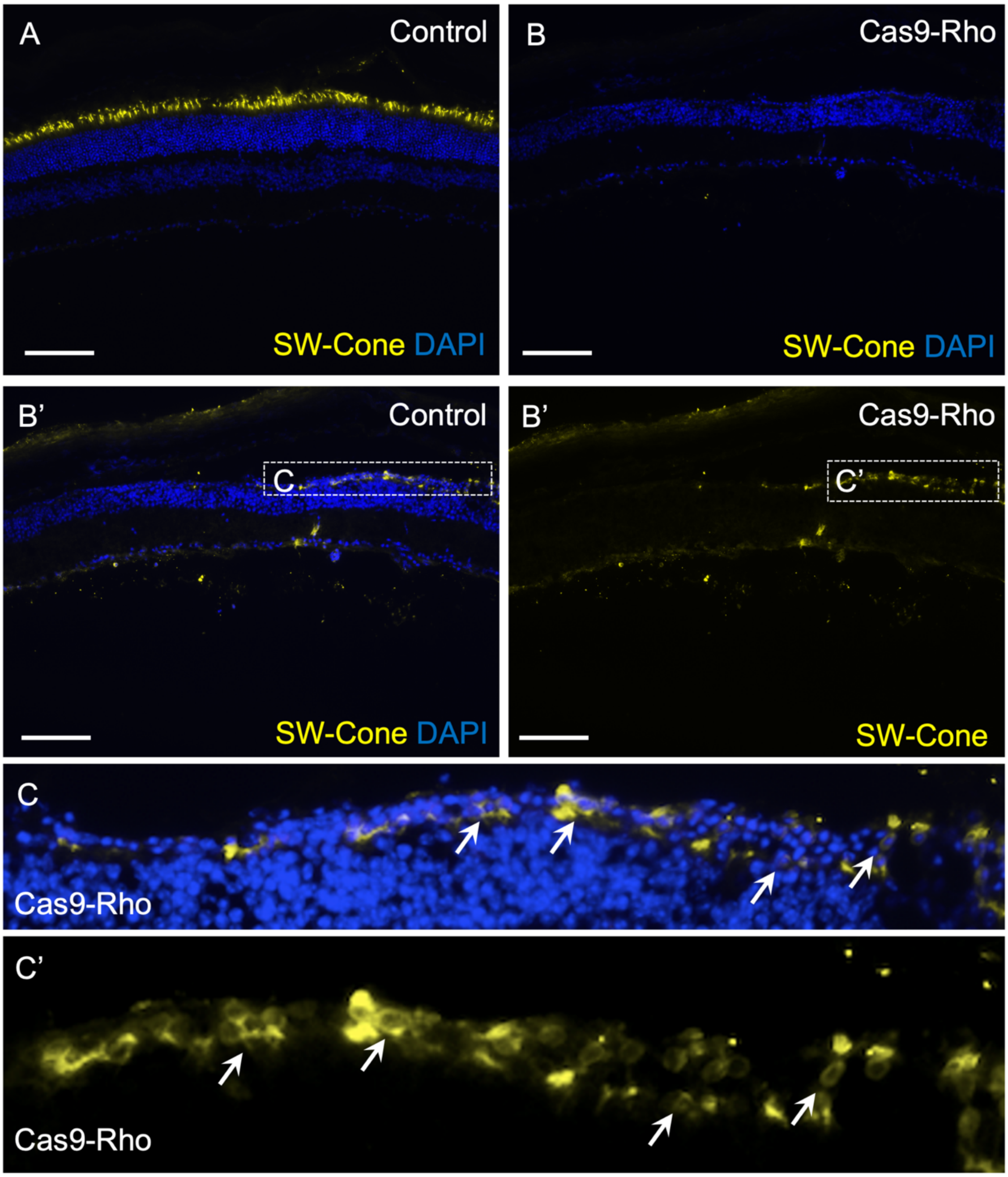

Supplementary figure S5. Dormant cones in CRISPR mouse model. (A) Control eye immunolabelled with short-wavelength cone-opsin (SW-Cone) (in yellow) and DAPI (in blue); (B) Cas9-Rho injected eye imaged with SW-Cone at the same intensity as panel A; (B') Cas9-Rho injected eye imaged with SW-Cone at the higher intensity; (C-C') Magnified parts from panel B'. Arrows point to cone cell bodies; Scale bars indicate 100µM.
